# Contribution of RaeB, a Putative RND-Type Transporter to Aminoglycoside and Detergent Resistance in *Riemerella anatipestifer*

**DOI:** 10.3389/fmicb.2017.02435

**Published:** 2017-12-08

**Authors:** Xin Zhang, Ming-Shu Wang, Ma-Feng Liu, De-Kang Zhu, Francis Biville, Ren-Yong Jia, Shun Chen, Kun-Feng Sun, Qiao Yang, Ying Wu, Xin-Xin Zhao, Xiao-Yue Chen, An-Chun Cheng

**Affiliations:** ^1^Institute of Preventive Veterinary Medicine, Sichuan Agricultural University, Chengdu, China; ^2^Avian Disease Research Center, College of Veterinary Medicine of Sichuan Agricultural University, Chengdu, China; ^3^Key Laboratory of Animal Disease and Human Health of Sichuan Province, Sichuan Agricultural University, Chengdu, China; ^4^Unité des Infections Bactériennes Invasives, Institut Pasteur, Paris, France

**Keywords:** *Riemerella anatipestifer*, *B739_0873* gene, *raeB* gene, RND efflux pump, aminoglycoside, detergent, resistance

## Abstract

*Riemerella anatipestifer* is an important pathogenic bacterium that infects ducks. It exhibits resistance to multiple classes of antibiotics. Multidrug efflux pumps play a major role as a mechanism of antimicrobial resistance in Gram-negative pathogens and they are poorly understood in *R. anatipestifer*. In this study, a gene encoding the B739_0873 protein in *R. anatipestifer* CH-1, which belongs to the resistance-nodulation-cell division (RND) efflux pump family, was identified. With respect to the substrate specificity of B739_0873, the antibiotic susceptibility testing showed that the *B739_0873* knockout strain was more sensitive to aminoglycosides and detergents than the wild-type strain. The transcription of *B739_0873* was up-regulated when *R. anatipestifer* CH-1 was exposed to sub-inhibitory levels of these substrates. From the gentamicin accumulation assay, we concluded that B739_0873 was coupled to the proton motive force to pump out gentamicin. Furthermore, site-directed mutagenesis demonstrated that Asp 400, Asp 401, Lys 929, Arg 959, and Thr 966 were the crucial function sites of B739_0873 in terms of its ability to extrude aminoglycosides and detergents. Finally, we provided evidence that *B739_0873* is co-transcribed with *B739_0872*, and that both *B739_0872* and *B739_0873* are required for aminoglycoside and detergent resistance. In view of these results, we designate B739_0873 as RaeB (*Riemerella anatipestifer* efflux).

## Introduction

*Riemerella anatipestifer* is a Gram-negative bacterium that belongs to the Flavobacteriaceae family (Segers et al., 1993). It infects ducks, geese, turkeys, chickens, and other birds, leads to contagious septicemia, and causes large economic losses in the duck industry (Ryll et al., 2001; Hess et al., 2013). Currently, at least 21 serotypes have been described worldwide, but there is no cross-protection between the different serotypes (Pathanasophon et al., 1996, 2002). Thus, antibiotics are still the major preventative and therapeutic approach against *R. anatipestifer* infection in poultry. Previous reports showed that the use of ceftiofur, novobiocin, penicillin, oxytetracycline, and streptomycin could reduce the mortality in ducks infected with *R. anatipestifer* (Sandhu and Dean, 1980; Chang et al., 2003). Unfortunately, the widespread use of antibiotics to treat *R. anatipestifer* infection has resulted in multidrug resistance in *R. anatipestifer*. Based on clinical investigation, *R. anatipestifer* is known to exhibit a very wide spectrum of drug resistance, including resistance to aminoglycosides, cephalosporins, lincosamides, macrolides, nalidixic acid, penicillins, rifampicin, and sulfonamides (Zhong et al., 2009; Sun et al., 2012). Using antibiotic therapy to achieve a good therapeutic effect has become more challenging. Thus, it is necessary to understand the multidrug resistance mechanisms of *R. anatipestifer* to find a way to prevent and treat *R. anatipestifer* infection.

In bacteria, multidrug efflux pumps are generally encoded by genetic elements capable of mediating intrinsic and acquired resistance to antibiotics (Li et al., 2015). Among these multidrug efflux pumps, the resistance-nodulation-cell division (RND) family members appear to be the most effective efflux systems in Gram-negative bacteria (Nikaido, 1994, 1996). RND transporters form a tripartite complex, consisting of an RND efflux pump, a periplasmic membrane fusion protein (MFP) and an outer membrane channel protein (OMP) to export the toxic compounds (Nikaido, 2011). To drive the export of the toxic substances, members of the RND family utilize the proton motive force (PMF) as the energy source (Paulsen et al., 1996). In recent years, numerous functions of RND efflux pumps have been identified; in addition to antibiotic extrusion, they are involved in bacterial pathogenicity and the bacterial stress response (Piddock, 2006). However, up to now, no reports have been published on the RND efflux pumps of *R. anatipestifer*.

In this study, we elucidated the biological functions of an RND efflux pump, the B739_0873 protein, namely RaeB for the first time, and obtained information to help to increase understanding of the multidrug-resistance mechanism of *R. anatipestifer*.

## Materials and Methods

### Bacterial Strains, Plasmids, and Growth Conditions

All the plasmids and strains used in this study are listed in **Table 1**. *R. anatipestifer* CH-1 (GenBank accession number: NC_018609) was isolated from the brains of diseased ducks from Chengdu, Sichuan, China, identified by our laboratory (Wang et al., 2014) and cultured in tryptic soybean broth (TSB, Oxoid) or tryptic soy agar (TSA, Oxoid) medium at 37°C in 5% CO_2_. *Escherichia coli* strains were grown in Luria-Bertani (LB, Oxoid) broth or LB agar (1.5% agar powder, Solarbio) at 37°C. The antimicrobial agents that were added to the media used for strain construction and selection were purchased from Sigma, and they were added to reach the following final concentrations: ampicillin (Amp), 100 μg/ml; cefoxitin (Cfx), 1 μg/ml; chloramphenicol (Cm), 25 μg/ml; kanamycin (Kan), 50 μg/ml; and spectinomycin (Spc), 80 μg/ml.

**Table 1:** Bacterial strains and plasmids used in this study.

Strains and plasmids	Description	Source or reference
**Strains**		
*R. anatipestifer* CH-1	Serotype 1, Cfx^S^, Kan^R^, Spc^S^	Wang et al., 2014
RA-CH-1 Δ*raeB*	*raeB* deletion mutant of *R. anatipestifer* CH-1 strain, Spc^R^	This study
RA-CH-1 Δ*raeB* pLMF03::*raeB*	RA-CH-1 Δ*raeB* carrying pLMF03::*raeB*, Kan^R^, Cfx^R^	This study
RA-CH-1 Δ*raeA* Δ*raeB*	*raeA-raeB* deletion mutant of *R. anatipestifer* CH-1 strain, Spc^R^	This study
RA-CH-1 Δ*raeA* Δ*raeB* pLMF03::*raeB*	RA-CH-1 Δ*raeA* Δ*raeB* carrying pLMF03::*raeB*, Kan^R^, Cfx^R^	This study
RA-CH-1 Δ*raeB* pD400A	RA-CH-1 Δ*raeB* carrying pLMF03::*raeB*_D400A_, Kan^R^, Cfx^R^	This study
RA-CH-1 Δ*raeB* pD401A	RA-CH-1 Δ*raeB* carrying pLMF03::*raeB*_D401A_, Kan^R^, Cfx^R^	This study
RA-CH-1 Δ*raeB* pK929E	RA-CH-1 Δ*raeB* carrying pLMF03::*raeB*_K929E_, Kan^R^, Cfx^R^	This study
RA-CH-1 Δ*raeB* pR959A	RA-CH-1 Δ*raeB* carrying pLMF03::*raeB*_R959A_, Kan^R^, Cfx^R^	This study
RA-CH-1 Δ*raeB* pT966E	RA-CH-1 Δ*raeB* carrying pLMF03::*raeB*_R966E_, Kan^R^, Cfx^R^	This study
*E. coli* K-12 X7232	*endA1 hsdR17*(*r_K_^-^ m_K_^+^*) *glnV44 thi-1 recA1 gyrA relA1*Δ(*lacZYA-argF*)*U169λpir deoR* (*Φ80dla*c Δ(*lacZ*)*M15*)	Roland et al., 1999
*E. coli* K-12 X7232 pRE112::*raeB*USD	*E. coli* K-12 X7232 carrying pRE112::*raeB*USD, Spc^R^, Cm^R^	This study
*E. coli* K-12 X7232 pRE112::*raeB-raeA*USD	*E. coli* K-12 X7232 carrying pRE112::*raeA-raeB*USD, Spc^R^, Cm^R^	This study
*E. coli* K-12 X7213	*thi-1 thr-1 leuB6 glnV44 fhuA21 lacY1 recA1 RP4-2-Tc*::*Mu λpir*Δ*asdA4*Δ*zhf-2*::*Tn10*	Roland et al., 1999
*E. coli* K-12 X7213 pRE112::*raeB*USD	*E. coli* K-12 X7213 carrying pRE112::*raeB*USD, Spc^R^, Cm^R^	This study
*E. coli* K-12 X7213 pRE112::*raeA-raeB*USD	*E. coli* K-12 X7213 carrying pRE112::*raeA-raeB*USD, Spc^R^, Cm^R^	This study
*E. coli* S17-1	*thi-1 thr leu tonA lac Y supE recA*::*RP4-2-Tc*::*Mu* Kan^R^	Miller and Mekalanos, 1988
*E. coli* S17-1 pLMF03::*raeB*	*E. coli* S17-1 carrying pLMF03::*raeB*, Amp^R^, Cfx^R^	This study
*E. coli* S17-1 pLMF03::*raeB*_D400A_	*E. coli* S17-1 carrying pLMF03::*raeB*_D400A_, Amp^R^, Cfx^R^	This study
*E. coli* S17-1 pLMF03::*raeB*_D401A_	*E. coli* S17-1 carrying pLMF03::*raeB*_D401A_, Amp^R^, Cfx^R^	This study
*E. coli* S17-1 pLMF03::*raeB*_K929E_	*E. coli* S17-1 carrying pLMF03::*raeB*_K929E_, Amp^R^, Cfx^R^	This study
*E. coli* S17-1 pLMF03::*raeB*_R959A_	*E. coli* S17-1 carrying pLMF03::*raeB*_R959A_, Amp^R^, Cfx^R^	This study
*E. coli* S17-1 pLMF03::*raeB*_T966E_	*E. coli* S17-1 carrying pLMF03::*raeB*_T966E_, Amp^R^, Cfx^R^	This study
*E. coli* BL21(DE3)	Expressing host cell	Invitrogen
*E. coli* BL21(DE3) pET32a (+)::*raeB*	*E. coli* BL21(DE3) carrying pET32a (+)::*raeB*, Amp^R^	This study
**Plasmids**		
pYES1	YAC-BAC shuttle plasmid, Spc^R^	Invitrogen
pRE112	*sac*B mobRP4 R6K ori, Cm^R^, pRE112-T-vector	Kong et al., 2011
pRE112::*raeB*USD	pRE112 carrying *raeB*USD from *R. anatipestifer* CH-1 and pYES1, Spc^R^, Cm^R^	This study
pRE112::*raeA-raeB*USD	pRE112 carrying *raeA-raeB*USD from *R. anatipestifer* CH-1 and pYES1, Spc^R^, Cm^R^	This study
pET32a (+)	pBR322 lacZ, IPTG-inducible promoter, Amp^R^	Invitrogen
pET32a (+)::*raeB*	pET32a (+) carrying the truncated *raeB* from *R. anatipestifer* CH-1, Amp^R^	This study
pLMF03	B739_0921 promoter, ori ColE1, ori pRA0726, Amp^R^, Cfx^R^	Liu et al., 2016
pLMF03::*raeB*	pLMF03 carrying *raeB* from *R. anatipestifer* CH-1, Cfx^R^	This study
pLMF03::*raeB*_D400A_	pLMF03 carrying *raeB* with mutation D400A, Cfx^R^	This study
pLMF03::*raeB*_D401A_	pLMF03 carrying *raeB* with mutation D401A, Cfx^R^	This study
pLMF03::*raeB*_K929E_	pLMF03 carrying *raeB* with mutation K929E, Cfx^R^	This study
pLMF03::*raeB*_R959A_	pLMF03 carrying *raeB* with mutation R959A, Cfx^R^	This study
pLMF03::*raeB*_T966E_	pLMF03 carrying *raeB* with mutation T966E, Cfx^R^	This study


*^R^, resistance; ^S^, sensitive; Amp, ampicillin; Cfx, cefoxitin; Cm, chloramphenicol; Kan, kanamycin; Spc, spectinomycin.*

### Polymerase Chain Reaction Method

The DNA fragments were obtained by polymerase chain reaction (PCR). PCR was carried out in a Hybaid PCR thermocycler (Bio-Rad) using the DNA Polymerase (Takara) according to manufacturer’s instructions. All primers used in this study were synthesized by Invitrogen.

### Quantitative Real-Time-PCR Analysis

To monitor the mRNA expression levels, quantitative real-time (qRT)-PCR was used. Total RNA was extracted from *R. anatipestifer* CH-1 strains in the logarithmic phase using RNAiso Plus (Takara) according to the manufacturer’s instructions. The RNA was then reverse transcribed into cDNA using the PrimeScript^TM^ RT Reagent Kit (Takara) with gDNA Eraser (Takara). PCR with SYBR^®^ Premix Ex Taq^TM^ II (Takara) was performed using the gene-specific primers P1-*16S rRNA*-F/P2-*16S rRNA*-R P3-*raeB*RT-F/P4-*raeB*RT-R and P5-*B739_0874*-F/P6-*B739_0874*-R. In the qRT-PCR analysis, 16S rRNA gene was used as an internal control. The relative gene expression was calculated using the 2^-Δ^ ^ΔCt^ method with Bio-Rad CFX Manager software (Schmittgen and Livak, 2008). Experiments were performed in triplicate.

### Construction of Knockout Strains

Recombinant suicide plasmids pRE112::*B739_0873*USD and pRE112::*B739_0872-B739_0873*USD were used to delete the *B739_0873* and both the *B739_0872* and *B739_0873* genes, respectively, by allelic exchange, according to previously described methods (Luo et al., 2015). Briefly, the primers P7-*raeB*up-F/P8-*raeB*up-R, P13-*raeA-raeB*up-F/P14-*raeA-raeB* up-R, and P11-*raeB*down-F/P12-*raeB*down-R were used to amplify the upstream and downstream homologous arm regions of the *B739_0873* and *B739_0872-B739_0873* genes in the *R. anatipestifer* CH-1 genome, respectively. The primers P9-*raeB*spc-F/P10-*raeB*spc-R and P15-*raeA-raeB*spc-F/P10-*raeB*spc-R were used to amplify Spc resistant (SpcR) cassettes from the plasmid pYES1. Each set of three PCR fragments was integrated using overlap PCR (Xiong et al., 2006) with primer pairs P7-*raeB*up-F/P12-*raeB*down-R and P13-*raeA-raeB*up-F/P12-*raeB*down-R. The fusion segments were then cloned into pRE112 to construct pRE112::*B739_0873*USD and pRE112::*B739_0872-B739_0873*USD. Subsequently, the recombinant plasmids were introduced into *R. anatipestifer* CH-1 by conjugation as described previously (Liao et al., 2015). The transconjugants were selected on TSA plates containing Spc (80 μg/ml) and Cfx (1 μg/ml), and they were then confirmed using PCR with the conserved 16S rRNA gene primers P1-*16S rRNA*-F/P2-*16S rRNA*-R and the corresponding identifying primers P16-*raeB*Ident-F/P17-*raeB*Ident-R and P18-*raeA-raeB*Ident-F/P19-*raeA-raeB*Ident-R. The resultant gene-deletion mutant strains were designated RA-CH-1 Δ*B739_0873* and RA-CH-1 Δ*B739_0872* Δ*B739_0873*.

### Construction of Complemented Strains

To confirm that the RA-CH-1 Δ*B739_0873* and RA-CH-1 Δ*B739_0872* Δ*B739_0873* phenotypes were due to the *B739_0873* gene deletion, the *E. coli–R. anatipestifer* shuttle plasmid pLMF03 was used to construct the recombinant plasmid pLMF03::*B739_0873* that contained an intact B739_0873 gene. Briefly, the primers P20-*raeB*-F/P21-*raeB*-R were used to amplify the *B739_0873* open reading frame (ORF), which was ligated into pLMF03 at *Nco*I and *Xho*I sites to generate pLMF03::*B739_0873*. For the complementation analysis, the pLMF03::*B739_0873* plasmid was introduced into the two *R. anatipestifer* CH-1 mutant strains by conjugation, as described previously (Wang et al., 2017). The transconjugants were selected on TSA plates containing Kan (50 μg/ml) and Cfx (1 μg/ml), and they were then confirmed using PCR with the conserved 16S rRNA gene primers P1-*16S rRNA*-F/P2-*16S rRNA*-R and the Cfx-identifying primers P24-*cfx*-F/P25-*cfx*-R. The resultant complemented mutant strains were designated RA-CH-1 Δ*B739_0873* pLMF03::*B739_0873* and RA-CH-1 Δ*B739_0872* Δ*B739_0873* pLMF03::*B739_0873*.

### Bacterial Growth Curves and Competition Experiments *in Vitro*

To evaluate the growth rates under non-competitive conditions, we monitored the growth curves for the wild-type strain, the RA-CH-1 Δ*B739_0873* mutant, and the RA-CH-1 Δ*B739_0873* complemented strain, according to the previously described method (Luo et al., 2015). *In vitro* competition experiments were performed with the wild-type strain and the RA-CH-1 Δ*B739_0873* mutant (Perez et al., 2012). Briefly, both strains were mixed in a 1:1 ratio when they were in the exponential phase. Subsequently, approximately 10^-5^ cells from the mixtures were added to 10 ml TSB and grown at 37°C. After 16 h, 10-fold serial dilutions of the cells were spread onto both TSA and TSA containing 80 μg/ml Spc in duplicate and incubated overnight at 37°C. The competition index (CI) was defined as the ratio between the number of mutant and wild-type CFUs. All experiments were performed in triplicate.

### Minimal Inhibitory Concentration Determination

A twofold serial dilution assay was used to measure the minimal inhibitory concentration (MIC) of antimicrobial agents for *R. anatipestifer* CH-1 strains according to the Clinical and Laboratory Standards Institute guidelines (CLSI, 2015). The general method from the CLSI document was used and there was no specific guidance document for *R. anatipestifer*. The *E. coli* American Type Culture Collection (ATCC) 25922 strain was used for quality control. Antimicrobial agents were serially diluted twofold in TSB broth with concentrations ranging between 1 and 512 μg/ml. The turbidity of the inoculum was adjusted to 10^7^ CFU/ml and 100 μl was added into every well. The 96-well microtiter plates were incubated at 37°C for 24 h. The lowest concentration that inhibited bacterial growth was considered to be the MIC. All tests were performed in triplicate.

### Gentamicin Accumulation Assay

To determine whether the B739_0873 protein was driven by PMF, the PMF inhibitor carbonyl cyanide *m*-chlorophenylhydrazone (CCCP, Sigma) was used to attempt to inhibit B739_0873 activity. First, a series of CCCP concentrations (1.25, 2.5, 5, and 10 μM) were prepared to determine the optimal concentration for the full survival of the wild-type strain, the RA-CH-1 Δ*B739_0873* mutant and the complemented strain RA-CH-1 Δ*B739_0873* pLMF03::*B739_0873*. Subsequently, the MICs of aminoglycosides and detergents for these *R. anatipestifer* CH-1 strains were determined with or without CCCP.

To further confirm that the B739_0873 protein was the inhibited efflux pump, a gentamicin accumulation assay was performed. Cells of *R. anatipestifer* CH-1, RA-CH-1 Δ*B739_0873* and RA-CH-1 Δ*B739_0873* pLMF03::*B739_0873* were grown in TSB broth and harvested at the exponential phase of growth, and then washed twice at room temperature with 50 mM phosphate buffer (pH 7.0), containing 1 mM MgSO_4_ and 0.4% (wt/vol) glucose. The washed cells were resuspended in the same buffer at a density of 1 mg (dry weight) per ml, including 32 μg/ml gentamicin. To determine the dry weight, cells harvested at the exponential phase of growth by centrifugation for 10 min at 8,000 rpm, removed the supernatant and dried to a constant weight at 60°C. At the 24-min time point, 5 μM CCCP was added to the cells suspensions to assess energy-dependent efflux. The samples were collected every 8 min, centrifuging at 12,000 *g* and 4°C for 1 min and the samples were immediately diluted into ice-cold phosphate buffer, followed by two washes and sonication on ice. Gentamicin uptake was measured using a Gentamicin ELISA Test Kit following the manufacturer’s instructions (Reagen).

### Site-Directed Mutagenesis

Subsequently, we investigated whether five conserved amino acid residues (Asp 400, Asp 401, Lys 929, Arg 959, and Thr 966) (Guan and Nakae, 2001; Su et al., 2006) in the *R. anatipestifer* CH-1 B739_0873 protein are vital for it to function. The point mutations were introduced into plasmid pLMF03::*B739_0873* using the overlap PCR method (Xiong et al., 2006). The mutant sites were designed in primers. As illustrated in **Table 2**, primer pairs P26-*raeB*_D400A_-R/P27-*raeB*_D400A_-F, P28-*raeB*_D401A_-R/P29- *raeB*_D401A_-F, P30-*raeB*_K929E_-R/P31-*raeB*_K929E_-F, P32-*raeB*_R959A_-R/P33-*raeB*_R959A_-F, and P34-*raeB*_T966E_-R/P35-*raeB*_T966E_-F, were reverse complementary. The upstream fragments were amplified from the *R. anatipestifer* CH-1 genome using primer pairs P20-*raeB*-F/P26-*raeB*_D400A_-R, P20-*raeB*-F/P28-*raeB*_D401A_, P20-*raeB*-F/P30-*raeB*_K929E_-R, P20-*raeB*-F/P32-*raeB*_R959A_-R, and P20-*raeB*-F/P34-*raeB*_T966E_-R, and the downstream fragments were amplified using primer pairs P27-*raeB*_D400A_-F/P21-*raeB*-R, P29-*raeB*_D401A_-F/P21-*raeB*-R, P31-*raeB*_K929E_-F/P21-*raeB*-R, P33-*raeB*_R959A_-F/P21-*raeB*-R, and P35-*raeB*_T966E_-F/P21-*raeB*-R. The two PCR fragments were integrated using overlap PCR with primer pair P20/P21 to generate versions of the B739_0873 gene, each with a single mutation site. The *B739_0873* mutant fragments were purified by MiniBEST DNA fragment Purification Kit (Takara) following the manufacturer’s instructions and then cloned into the pLMF03 plasmid to generate mutant recombinant plasmids, pLMF03::*B739_0873*_D400A_, pLMF03::*B739_0873*_D401A_, pLMF03::*B739_0873*_K929E_, pLMF03::*B739_0873*_R959A_, and pLMF03::*B739_0873*_T966E_. They were introduced into RA-CH-1 Δ*B739_0873* by conjugation, as described previously (Wang et al., 2017). The transconjugants were selected on TSA plates containing Kan (50 μg/ml) and Cfx (1 μg/ml), and they were then confirmed using PCR with the conserved 16S rRNA gene primers P1-*16S rRNA*-F/P2-*16S rRNA*-R and the Cfx-identifying primers P24-*cfx*-F/P25-*cfx*-R. The resultant complemented mutant strains were designated RA-CH-1 Δ*B739_0873* pD400A, RA-CH-1 Δ*B739_0873* pD401A, RA-CH-1 Δ*B739_0873* pK929E, RA-CH-1 Δ*B739_0873* pR959A, and RA-CH-1 Δ*B739_0873* pT966A.

**Table 2:** Primers used in this study.

Primer	Sequence (5′–3′)	Source
P1-*16S rRNA*-F	CGAAAGTGATAAGTTAGCCACCT	This study
P2-*16S rRNA*-R	GCAGCACCTTGAAAATTGTCC	This study
P3-*raeB*RT-F	AAGAGCCTTCGTTATCACAGT	This study
P4-*raeB*RT-R	AATTTCTCGCTCTTGCCCTC	This study
P5-*B739_0874*-F	TCACACGAATACAATGGTT	This study
P6-*B739_0874*-R	AGGCTGTACTTTGATAACTCT	This study
P7-*raeB*up-F	CGCGGATCCCACTACAACTAGATCAAGCG	This study
P8-*raeB*up-R	AATAAGGGCGACACGGAAATGTTAATGGCGTTGATTTTCCTT	This study
P9-*raeB*spc-F	AAGGAAAATCAACGCCATTAACATTTCCGTGTCGCCCTTATT	This study
P10-*raeB*spc-R	ATCTTCCTTAGCCAGTTTTCTGAGGCCATCAAACCACGTCA	This study
P11-*raeB*down-F	TGACGTGGTTTGATGGCCTCAGAAAACTGGCTAAGGAAGAT	This study
P12-*raeB*down-R	CGGGGTACCACCTACGATATGACGGTTC	This study
P13-*raeA-raeB*up-F	CGCGGATCCGCCGTCTGTACTTATTTCG	This study
P14-*raeA-raeB*up-R	AATAAGGGCGACACGGAAATGTTTATTTTTCTGTTAAAGTTCT	This study
P15-*raeA-raeB*spc-F	AGAACTTTAACAGAAAAATAAACATTTCCGTGTCGCCCTTATT	This study
P16-*raeB*Ident-F	GGATCCATGAATAAAAAAACATTATTATCTATTATAG	This study
P17-*raeB*Ident-R	TAACTTTGTTTTAGGGCGACT	This study
P18-*raeA-raeB*Ident-F	TAGACAGGCTTATCTTGGACA	This study
P19-*raeA-raeB*Ident-R	TAACAAAATACCATCAAGGCTA	This study
P20-*raeB*-F	CATGCCATGGATGAAATTAGCAGAAGTATCC	This study
P21-*raeB*-R	CCGCTCGAGCTCCATTTTTTGAAGCCTCT	This study
P22-Truncated *raeB*-F	CGCGGATCCATGACCATATATCCAGGGGCATC	This study
P23-Truncated *raeB*-R	CCGCTCGAGGTTCTGTTGATTGATACGAGCA	This study
P24-*cfx*-F	CTCGCCAGAATCATAGACAAG	This study
P25-c*fx*-R	ATAGCGCATAAGACAGGTTC	This study
P26-*raeB*_D400A_-R	CACAATGGCATCAGCTACCAATATCCCT	This study
P27-*raeB*_D400A_-F	AGGGATATTGGTAGCTGATGCCATTGTG	This study
P28-*raeB*_D401A_-R	CACAATGGCGGCGTCTACCAATATCCCT	This study
P29-*raeB*_D401A_-F	AGGGATATTGGTAGACGCCGCCATTGTG	This study
P30-*raeB*_K929E_-R	GCATTTTCCGCCACCAAACCAATCAAC	This study
P31-*raeB*_K929E_-F	GTTGATTGGTTTGGTGGCGGAAAATGC	This study
P32-*raeB*_R959A_-R	CATCAAAATAGGACGAAGAGCAGC	This study
P33-*raeB*_R959A_-F	GCTGCTCTTCGTCCTATTTTGATG	This study
P34-*raeB*_T966E_-R	CCATCGCTATCGTTTCCATCAAAA	This study
P35-*raeB*_T966E_-F	TTTTGATGGAAACGATAGCGATGG	This study


*^-^, mutation sites underlined.*

### Antibody Preparation

The recombinant plasmid pET32a (+)::*B739_0873* was used to express B739_0873 protein. Briefly, the primers P22-Truncated *raeB*-F/P23-Truncated *raeB*-R were used to amplify the truncated B739_0873 ORF, which was ligated into pET32a (+) at *Bam*HI and *Xho*I sites to generate pET32a (+)::*B739_0873*.

Strain *E. coli* BL21 (DE3) pET32a (+)::*B739_0873* was grown overnight at 37°C in LB broth containing 100 μg/ml Amp. Subsequently, the LB broth containing 100 μg/ml Amp was inoculated with the overnight culture to an optical density at 600 nm (OD_600_) of 0.05 and grown at 37°C. Expression was induced by adding 0.6 mM isopropyl β-D-1-thiogalactopyranoside (IPTG) at an OD_600_ of 0.6 for 6 h at 37°C. Cells harvested by centrifugation for 10 min at 8,000 rpm and 4°C were suspended in lysis buffer (20 mM Tris–HCl, pH 8.0) and then sonicated on ice. After centrifugation at 10,000 rpm and 4°C for 10 min, the supernatant of the lysate was loaded onto an equilibrated (with 20 mM Tris–HCl, pH 8.0) nickel-nitrilotriacetic acid (NTA) column (7sea Biotech, China). By washing with lysis buffer (20 mM Tris–HCl, pH 8.0) containing 20 and 50 mM imidazole, non-specific contaminants were removed. Subsequently, the B739_0873 protein was eluted with lysis buffer (20 mM Tris–HCl, pH 8.0) containing 200 mM imidazole. Then the purified protein was placed in the dialysis membrane (Solarbio), soaked in phosphate-buffered saline (PBS) buffer (pH 7.4) and changed the buffer every other 1, 2, 4 h, the final dialyzed overnight, to eliminate any residual imidazole.

The purified B739_0873 His-tagged protein was used to generate a polyclonal antibody. Approximately 2 mg of B739_0873 protein emulsified in complete Freund’s adjuvant (Sigma) was used to immunize four rabbits (the local rabbit industry in Ya’an, Sichuan) via intradermal injections. Subsequently, booster doses of 3 and 4 mg of B739_0873 protein were prepared in incomplete Freund’s adjuvant (Sigma), and the immunization was given after 2 and 3 weeks, respectively, using subcutaneous injections. A week after the last immunization, the antibody was collected from the ear vein of the rabbits and frozen at -80°C.

### Immunoblot Analysis

Strains RA-CH-1 Δ*B739_0873* pLMF03, RA-CH-1 Δ*B739_0873* pLMF03::*B739_0873*, RA-CH-1 Δ*B739_0873* pD400A, RA-CH-1 Δ*B739_0873* pD401A, RA-CH-1 Δ*B739_0873* pK929E, RA-CH-1 Δ*B739_0873* pR959A, and RA-CH-1 Δ*B739_0873* pT966A were grown at 37°C in TSB containing 1 μg/ml Cfx. Bacteria were harvested by centrifugation until they grew to exponential phase, suspended in the PBS buffer and then sonicated on ice. The sonicated cells were suspended in loading buffer and heated for 5 min at 100°C. Proteins were separated by 12% sodium dodecyl sulfate polyacrylamide gel electrophoresis (SDS-PAGE) using TGX Stain-Free^TM^ FastCast^TM^ Acrylamide Kit (Bio-Rad) and subsequently transferred to a polyvinylidene difluoride (PVDF) membrane (Bio-Rad). Non-specific binding sites were blocked with 5% skim milk (Solarbio) in TBS-Tween 20 (0.05%). The PVDF membranes were probed with B739_0873 rabbit polyclonal antibody (1:200), followed by a 1:3,000 dilution of a goat anti-rabbit IgG alkaline phosphatase-conjugated secondary antibody (Bio-Rad). The binding of antibodies to protein was revealed using a substrate for horseradish peroxidase (HRP)-based chemiluminescence Western blot detection following the manufacturer’s instructions (Takara).

### Ethics Statement

All animals were handled in strict accordance with good animal practices, as defined by the local animal welfare bodies. The protocol for the animal work to be performed at Sichuan Agriculture University was reviewed and approved by the Sichuan Agriculture University ethics committee in September 2014.

### Statistical Analysis

Statistical analysis was performed using GraphPad Prism version 7 software for Windows. The significance of the between-group differences was ascertained using Student’s *t*-test. A value of *P* < 0.05 was considered significant.

## Results

### Sequence Analysis of *R. anatipestifer* CH-1 *raeB*

The *B739_0873* gene of *R. anatipestifer* CH-1 consists of an ORF with 3,156 bp, which encodes a 1,051-amino acid protein with a putative molecular mass of 115 kDa. The B739_0873 protein is annotated as a cation/multidrug efflux pump in the National Center for Biotechnology Information (NCBI) database. A protein–protein Basic Local Alignment Search Tool (BLASTP) analysis of the B739_0873 amino acid sequence of different *R. anatipestifer* strains indicated 99–100% identity. Homologs of B739_0873 are also present in *Riemerella columbina*, *Riemerella columbipharyngis*, *Flavobacteriaceae bacterium 3519-10*, *Salinimicrobium terrae*, *Chryseobacterium treverense*, *Chryseobacterium solincola*, *Salegentibacter salegens*, and *Salegentibacter agarivorans*, and identities range from 77 to 83%. However, the function of all these homologs has not previously been characterized.

Further analysis of the B739_0873 protein revealed that it was located in the cytoplasmic membrane and there were 12 trans-membrane domains (TMD) and two extremely large periplasmic loops between helix 1 and 2 and between helix 7 and 8. These results are consistent with the general features of RND efflux pumps (Tseng et al., 1999). The Clustal comparison showed that B739_0873 shares 28% identity with *E. coli* AcrB and 27% identity with *Pseudomonas aeruginosa* MexB. Furthermore, the essential residues in *E. coli* AcrB (Asp 407, Asp 408, Lys 940, Arg 971, and Thr 978) (Su et al., 2006; Takatsuka and Nikaido, 2006) and *P. aeruginosa* MexB (Asp 407, Asp 408, Lys 939, Arg 969, and Thr 976) (Guan and Nakae, 2001) that are responsible for the proton relay pathway are well conserved in B739_0873 (corresponding to Asp 400, Asp 401, Lys 929, Arg 959, and Thr 966).

**FIGURE 1:**
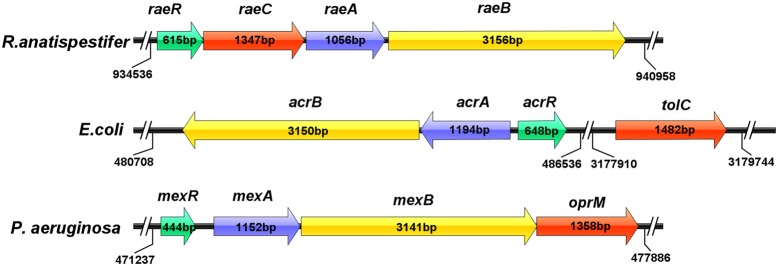
Schematic representation of the position and size of the resistance-nodulation-cell division (RND) efflux pump genes in the genomes of *R. anatipestifer* CH-1, *E. coli*, and *P. aeruginosa*. The RND efflux pump genes are described in yellow, membrane fusion protein (MFP) in blue, the outer membrane protein (OMP) in red, and the regulator in green.

Analysis of the upstream regions of the *B739_0873* gene showed that the *B739_0872* gene encodes a protein belonging to the MFP family (Dinh et al., 1994), the *B739_0871* gene encodes a protein belonging to the outer membrane factor (OMF) family (Paulsen et al., 1997) and the *B739_0870* gene encodes a tetracycline resistance repressor protein (TetR) family transcriptional regulator (Ramos et al., 2005). These three genes occur together with the *B739_0873* gene on the *R. anatipestifer* CH-1genome (**Figure 1**). Sequence alignments showed that B739_0870 shares 31% identity with *E. coli* AcrR, B739_0872 shares 24% identity with *E. coli* AcrA and 25% identity with *P. aeruginosa* MexA, and B739_0871 shares 20% identity with *E. coli* TolC and 23% identity with *P. aeruginosa* OprM.

Overall, the bioinformatic analysis suggests that B739_0873 is a putative RND efflux pump. Thus, we designated the *B739_0870-0871-0872-0873* genes as *raeR-raeC-raeA-raeB* and the B739_0870-0871-0872-0873 proteins as RaeR-RaeC-RaeA-RaeB described below.

### Increased Aminoglycoside and Detergent Susceptibility of *raeB* Mutant

To investigate the role of *raeB*, a mutated *raeB* was constructed in *R. anatipestifer* CH-1. To determine whether the inactivation of *raeB* affected the growth state of *R. anatipestifer* CH-1, both the growth curves and the CI values were evaluated. The results showed that RA-CH-1 Δ*raeB* had the same growth rate and CI value as the wild-type strain (**Supplementary Figures S1**, **S2**). To determine whether the inactivation of *raeB* affected the virulence of *R. anatipestifer* CH-1, the median lethal dose (LD_50_) was measured as described previously by Wang et al. (2017). The results showed that there were no significant LD_50_ changes between the wild-type strain and RA-CH-1 Δ*raeB* (data not shown).

In a second set of experiments, the susceptibility of the RA-CH-1 Δ*raeB* mutant and the wild-type strain to a variety of antimicrobial agents with dissimilar structures (including Amp, acriflavine, azithromycin, aztreonam, carbenicillin, cefradine, ceftiofur, cefuroxime, cephalothin, chloromycetin, ciprofloxacin, enrofloxacin, erythromycin, ethidium bromide, florfenicol, gentamicin, Kan, lincomycin, nalidixic acid, rifampicin, sodium dodecyl sulfate (SDS), streptomycin, sulfamethoxazole, tetracycline, trimethoprim, Triton X-100, and vancomycin) was compared. It was shown that deletion of *raeB* increased susceptibility to all the aminoglycosides tested [streptomycin (eightfold), Kan (eightfold), and gentamicin (16-fold)] and all the detergents tested [Triton X-100 (fourfold) and SDS (16-fold)] (**Table 3**). There were no MIC changes between the wild-type strain and the mutant strain for any of the tested cephalosporins, chloramphenicols, cationic dyes, quinolones, glycopeptides, lincosamides, macrolides, penicillins, rifampicin, sulfonamides, and tetracyclines (Supplementary Table S1). To exclude the possibility that gene inactivation had a polar effect on the transcription of adjacent genes, RT-PCR was performed to measure the mRNA levels of the downstream gene, *B739_0874*, and it was shown that there was no significant difference in *B739_0874* gene transcription between the wild-type strain and the RA-CH-1 Δ*raeB* mutant (data not shown). This result revealed that the changes were caused solely by *raeB*. Complementation of the RA-CH-1 Δ*raeB* mutant with plasmid pLMF03::*raeB* restored resistance to aminoglycosides and detergents (**Table 3**). This result indicated that *raeB* is involved in aminoglycoside and detergent resistance.

**Table 3:** MICs of aminoglycosides and detergents for *R. anatipestifer* CH-1 strains.

Strains	MIC (μg/ml)				
	
	Gentamicin	Streptomycin	Kanamycin	Sodium dodecyl sulfate	Triton X-100
*R. anatipestifer* CH-1	32	128	256	640	160
RA-CH-1 Δ*raeB*	2	16	32	40	40
RA-CH-1 Δ*raeB* pLMF03::*raeB*	32	128	256	640	160
RA-CH-1 Δ*raeA* Δ*raeB*	2	16	32	40	40
RA-CH-1 Δ*raeA* Δ*raeB* pLMF03::*raeB*	2	16	32	40	40
RA-CH-1 Δ*raeB* pD400A	2	16	32	40	40
RA-CH-1 Δ*raeB* pD401A	2	16	32	40	40
RA-CH-1 Δ*raeB* pK929E	2	16	32	40	40
RA-CH-1 Δ*raeB* pR959A	2	16	32	40	40
RA-CH-1 Δ*raeB* pT966E	2	16	32	40	40


### Induction of *raeB* Transcription by Aminoglycoside and Detergent Exposure

**FIGURE 2:**
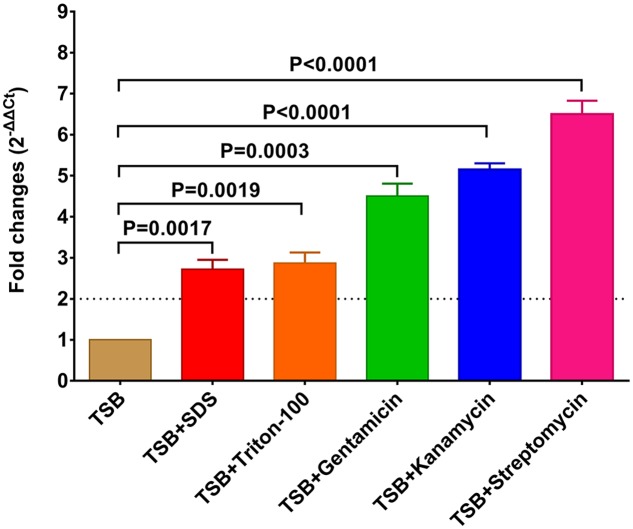
Relative fold changes of *raeB* mRNA expression levels in *R. anatipestifer* CH-1 after treatment with aminoglycosides and detergents. The sub-inhibitory concentrations of aminoglycosides and detergents were added to the TSB broth (gentamicin, 16 μg/ml; Kan, 128 μg/ml; streptomycin, 64 μg/ml; Triton X-100, 80 μg/ml; SDS, 320 μg/ml). SDS, sodium dodecyl sulfate. Error bars indicate the standard deviation (*n* = 3).

To address whether *raeB* is regulated by aminoglycosides and detergents, sub-inhibitory concentrations of aminoglycosides and detergents were added to an *R. anatipestifer* CH-1 growth culture and the transcription of *raeB* was measured using qRT-PCR. As shown in **Figure 2**, the level of *raeB* expression was up-regulated by two- to sevenfold after treatment with Triton X-100, SDS, streptomycin, Kan, or gentamicin.

### Inhibition of RaeB Activity by CCCP

The final concentration of CCCP in TSB broth was 5 μM, which did not affect the growth of the wild-type strain, the RA-CH-1 Δ*B739_0873* mutant and the complemented strain RA-CH-1 Δ*B739_0873* pLMF03::*B739_0873*. The results showed that the addition of CCCP (5 μM) decreased the MICs of streptomycin (eightfold), gentamicin (16-fold), and Kan (eightfold) for the wild-type strain and RA-CH-1 Δ*raeB* pLMF03::*raeB*. Similarly, the MICs of SDS and Triton X-100 for the wild-type strain and RA-CH-1 Δ*raeB* pLMF03::*raeB* decreased by 4- and 16-fold, respectively, in the presence of CCCP. In contrast, CCCP addition did not modify the MICs of streptomycin, gentamicin, Kan, SDS, and Triton X-100 for RA-CH-1 Δ*raeB*. The MICs of streptomycin, gentamicin, Kan, SDS, and Triton X-100 for the wild-type strain and RA-CH-1 Δ*raeB* pLMF03::*raeB* in the presence of CCCP were consistent with those evidenced for RA-CH-1 Δ*raeB* in the absence or presence of CCCP. These data indicated that an aminoglycoside and detergent efflux pump exists in *R. anatipestifer* CH-1.

**FIGURE 3:**
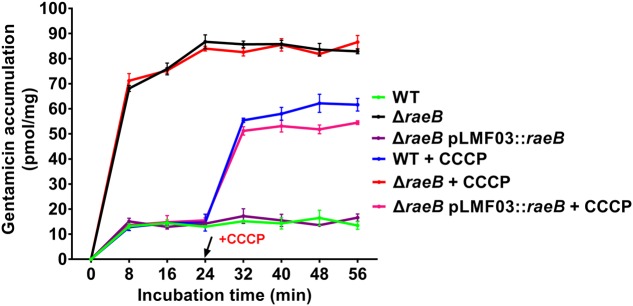
Accumulation of gentamicin by *R. anatipestifer* CH-1 cells. CCCP, carbonyl cyanide *m*-chlorophenylhydrazone; WT, *R. anatipestifer* CH-1; Δ*raeB*, RA-CH-1 Δ*raeB*; Δ*raeB* pLMF03::*raeB*, RA-CH-1 Δ*raeB* pLMF03::*raeB* complemented strain. Error bars indicate the standard deviation (*n* = 3). The arrow indicate CCCP was added at 24-min.

As shown in **Figure 3**, at the 24-min incubation time point, the accumulation level of gentamicin achieved a steady-state for all strains. The gentamicin accumulation level in the mutant strain RA-CH-1 Δ*raeB* was about six times higher than that in the wild-type strain and RA-CH-1 Δ*raeB* pLMF03::*raeB*. After CCCP was added to the cells containing gentamicin at 24-min, the accumulation of gentamicin increased in the wild-type strain and RA-CH-1 Δ*raeB* pLMF03::*raeB*; these accumulation levels were lower than that in RA-CH-1 *raeB*. In contrast, under our conditions, CCCP had no significant effect on the level of gentamicin accumulation in the mutant strain RA-CH-1 Δ*raeB*. In addition, the wild-type strain, RA-CH-1 Δ*raeB*, and RA-CH-1 Δ*raeB* pLMF03::*raeB* with no CCCP added at 24-min served as controls. These data indicated that RaeB pumped out gentamicin in an energy-dependent process, presumably coupled to the PMF.

### Identification of the Functional Sites in RaeB

**FIGURE 4:**
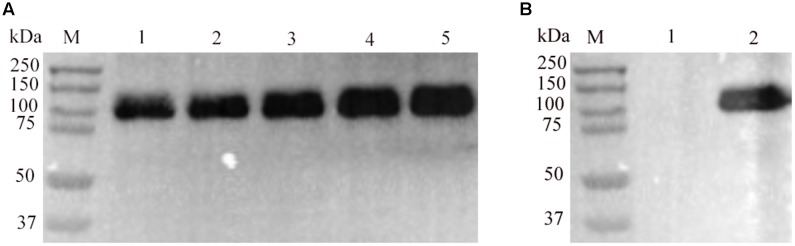
Immunoblotting analysis of RaeB protein and mutant RaeB proteins produced from pLMF03 in RA-CH-1 Δ*raeB*. **(A)** Lanes consist of molecular size marker (Bio-Rad) (M), RA-CH-1 Δ*raeB* pD400A (1), RA-CH-1 Δ*raeB* pD401A (2), RA-CH-1 Δ*raeB* pK929E (3), RA-CH-1 Δ*raeB* pR959A (4), and RA-CH-1 Δ*raeB* pT966E (5). **(B)** Lanes consist of molecular size marker (M), RA-CH-1 Δ*raeB* pLMF03 (1), and RA-CH-1 Δ*raeB* pLMF03::*raeB* (2).

We investigated whether amino acid residues Asp 400, Asp 401, Lys 929, Arg 959, and Thr 966 are vital for the function of the RaeB protein of *R. anatipestifer* CH-1, five mutant recombinant plasmids, pLMF03::*raeB*_D400A_, pLMF03::*raeB*_D401A_, pLMF03::*raeB*_K929E_, pLMF03::*raeB*_R959A_, and pLMF03::*raeB*_T966E_, were constructed. These plasmids were introduced into RA-CH-1 ΔB739_0873, and the MICs for RA-CH-1 Δ*raeB* pD400A, RA-CH-1 Δ*raeB* pD401A, RA-CH-1 Δ*raeB* pK929E, RA-CH-1 Δ*raeB* pR959A, and RA-CH-1 Δ*raeB* pT966A were examined. It was shown that none of these mutant strains exhibited restored resistance to aminoglycosides and detergents (**Table 3**). To exclude the possibility that these results occurred because these mutant *raeB* genes were not expressed, we detected the expression of these mutant proteins by immunoblotting with antibody against the RaeB protein. The immunoblot analysis showed that all these RaeB mutant proteins expressed in RA-CH-1 Δ*raeB* (**Figure 4A**) were detectable and indistinguishable from the RaeB protein expressed in RA-CH-1 Δ*raeB* (**Figure 4B**).

### Requirement of *raeA* and *raeB* for Aminoglycoside and Detergent Resistance

We confirmed that *raeB* is co-transcribed with *raeA* (**Supplementary Figure S3**) as previously reported by Liu et al. (2017). To understand more about *raeB*, the RA-CH-1 Δ*raeA* Δ*raeB* mutant was constructed. Antibiotic susceptibility testing showed that this mutant displayed MICs that were decreased by eightfold for streptomycin, 16-fold for gentamicin, eightfold for Kan, 16-fold for SDS, and fourfold for Triton X-100 (**Table 3**). However, complementation of the RA-CH-1 Δ*raeA* Δ*raeB* mutant with plasmid pLMF03::*raeB* did not restore resistance to any of these compounds (**Table 3**). These results indicate that both *raeA* and *raeB* are required for aminoglycoside and detergent resistance.

## Discussion

RND efflux pumps are widespread among Gram-negative bacteria and play an important role in producing multidrug resistance (Nikaido, 2011). They are located in the cytoplasmic membrane, capturing structurally and functionally dissimilar substrates either from the cytoplasm or from the periplasmic space and then transporting them to the extracellular medium (Nikaido, 2011). As reported, there are total six RND efflux pumps in *E. coli* (Anes et al., 2015) and 10–12 in *P. aeruginosa* (Poole, 2008; Fernando and Kumar, 2013). Among these RND efflux pumps, AcrB of *E. coli* and MexB of *P. aeruginosa* are the best studied members (Puzari and Chetia, 2017). Here, genome sequence analysis suggested that the *raeB* gene in *R. anatipestifer* CH-1 encodes a putative RND efflux pump, and we investigated the biological function of RaeB in *R. anatipestifer* CH-1, as well as its energy source and functional site.

As is well known, RND efflux pumps are responsible for the extrusion of a very wide range of antimicrobial agents (Nikaido, 2011). The substrate specificity study revealed that inactivation of the *raeB* gene in *R. anatipestifer* CH-1 decreased resistance to the aminoglycosides and detergents. According to previous studies, aminoglycoside efflux pumps have been identified in many bacteria; well-characterized examples include AcrD in *E. coli* (Rosenberg et al., 2000) and MexY in *P. aeruginosa* (Morita et al., 2012). Comparing the substrate specificity of these three efflux pumps, it is clear that they all export aminoglycosides and detergents. Although there were no MIC changes between the wild-type strain and the RA-CH-1 Δ*raeB* mutant for quinolones and β-lactams, it was still not sure that whether RaeB exported quinolones and β-lactams or not because other resistance factors could compromise RaeB-mediated resistance to quinolones and β-lactams (Morita et al., 2001). The substrate specificity of *E. coli* AcrD is determined predominantly by the two large periplasmic loops (Elkins and Nikaido, 2002). In *P. aeruginosa* MexY, a region corresponding to a proximal binding pocket connected to a periplasm-linked cleft, part of a drug export pathway of *E. coli* AcrB, was identified and proposed to play a role in aminoglycoside recognition (Lau et al., 2014). Thus, it was speculated that the periplasmic loops between helix 1 and 2 and between helix 7 and 8 and the corresponding aminoglycoside recognition region of MexY in RaeB are related to its substrate specificity.

According to the reports, the expression of efflux pump genes is induced by the addition of the export substrate (Guglierame et al., 2006; Morita et al., 2006; Pletzer and Weingart, 2014). Similarly, it was found that *raeB* in *R. anatipestifer* CH-1 was up-regulated in the presence of aminoglycosides and detergents. In Gram-negative bacteria, regulatory proteins can interact with inducers and therefore increase the transcription of efflux pump genes (Ramos et al., 2005). Usually, the regulatory protein genes are located next to the efflux pump genes in the chromosome (Cuthbertson and Nodwell, 2013). In *E. coli*, *acrR* is located in a region downstream of *acrB* and AcrR functions as a repressor of the AcrB efflux pump (Ma et al., 1996). Not surprisingly, the *raeR* gene, which is located in a region upstream of *raeB* in *R. anatipestifer* CH-1, encodes a TetR family transcriptional regulator. This finding is meaningful for future research on the regulatory mechanism underlying *R. anatipestifer* resistance.

It has been demonstrated that that AcrB of *E. coli* utilizes PMF as energy for its transport function (Paulsen et al., 1996). In this study, RaeB of *R. anatipestifer* CH-1 was proved to transport gentamicin by PMF. Additionally, in AcrB of *E. coli*, five charged residues Asp 407, Asp 408, Lys 940, Arg 971, and Thr 978 produce a proton-relay network, and the protonation and deprotonation of the residues disturbs the network and initiates a series of conformational changes that result in substrate transport (Su et al., 2006). We also determined that these corresponding amino acid residues in RaeB of *R. anatipestifer* CH-1 (Asp 400, Asp 401, Lys 929, Arg 959, and Thr 966) played an essential role in aminoglycoside and detergent transport. However, no structural information about the RaeB protein is currently available, so further studies determining whether these five amino acid residues are relevant to the proton transport process in *R. anatipestifer* CH-1 are needed.

In some Gram-negative bacteria, RND efflux pumps have a role not only in antibiotic resistance, but also in bacterial fitness and virulence (Alvarez-Ortega et al., 2013). As reported for *Enterobacter cloacae*, growth curves and competition experiments *in vitro* demonstrate that lack of the AcrB efflux pump poses a fitness cost to *E. cloacae* (Perez et al., 2012). However, deleting the *raeB* gene did not alter the fitness of *R. anatipestifer* CH-1. This may reflect a possible adaptation, in that other efflux pump systems may exist in *R. anatipestifer* CH-1 and were overproduced to compensate for the deletion. Additionally, efflux pumps AcrB in *E. cloacae* (Perez et al., 2012), AcrB in *Klebsiella pneumoniae* (Padilla et al., 2010), VexM and VexF in *Vibrio cholerae* (Bina et al., 2008), MmpL11 in *Mycobacterium tuberculosis* (Tullius et al., 2011), and AcrB in *Salmonella typhimurium* (Buckley et al., 2006) contribute to bacterial virulence. Interestingly, in this study, LD_50_ was measured between the wild-type strain and the mutant strain RA-CH-1 Δ*raeB*, the result showed that deletion of the *raeB* gene had no significant impact on the LD_50_ of *R. anatipestifer* CH-1. The possibility that *raeB* is not the main virulence factor in *R. anatipestifer* CH-1 may account for this result. All these data suggest that RND efflux pumps have multiple different physiological functions in different species of bacteria.

## Author Contributions

A-CC and XZ conceived of and designed the project. XZ and M-FL constructed the *raeB* and *raeA-raeB R. anatipestifer* CH-1 deletion mutants and assessed their antimicrobial resistance. D-KZ and XZ assessed the mRNA levels of the *raeB* and *B739_0874* genes using RT-PCR. XZ, M-SW, and M-FL constructed the mutant recombinant plasmids, pLMF03:: *raeB*D400A, pLMF03::*raeB*D401A, pLMF03::*raeB*K929E, pLMF03::*raeB*R959A, and pLMF03::*raeB*T966E. XZ and M-FL constructed the RA-CH-1 Δ*raeB* complemented strain and the RA-CH-1 Δ*raeA* Δ*raeB* complemented strain. XZ, QY, and YW performed the CCCP inhibition assay and gentamicin accumulation assay. M-SW, K-FS, and X-YC constructed the bacterial growth curves and performed the *in vitro* competition experiments. X-XZ, R-YJ, and SC performed the LD_50_ determination for *R. anatipestifer* CH-1 and RA-CH-1 Δ*raeB*. XZ, A-CC, and FB drafted and revised the manuscript. All the authors read and approved the final version of the manuscript.

## Conflict of Interest Statement

The authors declare that the research was conducted in the absence of any commercial or financial relationships that could be construed as a potential conflict of interest.

## Supplementary Material

**FIGURE S1 |** Growth curves for *R. anatipestifer* CH-1 strains. WT, *R. anatipestifer* CH-1; Δ*raeB*, RA-CH-1 Δ*raeB*; Δ*raeB* pLMF03::*raeB*, RA-CH-1 Δ*raeB* pLMF03::*raeB* complemented strain. Error bars indicate the standard deviation (*n* = 3).

**FIGURE S2 |** Competition experiments *in vitro* for the wild-type strain *R. anatipestifer* CH-1 and the RA-CH-1 ΔB739_0873 mutant. The mixed cells in the exponential phase were diluted to 10^-6^, 10^-7^, 10^-8^, and 10^-9^ and then spread onto both TSA and TSA containing 80 μg/ml spectinomycin and incubated overnight at 37°C. The competition index (CI) was defined as the ratio between the number of mutant and wild-type CFUs. Error bars indicate the standard deviation (*n* = 3).

**FIGURE S3 |** PCR of the *raeA-raeB* truncated fragment using different templates. The lanes consist of a molecular size marker (Takara) (M), a positive-control with the *R. anatipestifer* CH-1 genome DNA as the template (1), a negative-control with H_2_O as the template (2), a positive-control with total RNA of *R. anatipestifer* CH-1 before removing the DNA as the template (3), a negative-control with total RNA of *R. anatipestifer* CH-1 after removing the DNA as the template (4), and cDNA of *R. anatipestifer* CH-1 as the template (5).
