# Supplementary figures and images for: Contribution of RaeB, a Putative RND-Type Transporter to Aminoglycoside and Detergent Resistance in Riemerella anatipestifer
Source: Front Microbiol. 2017 Dec 8;8:2435. doi: 10.3389/fmicb.2017.02435 (PMC5727081; doi:10.3389/fmicb.2017.02435)

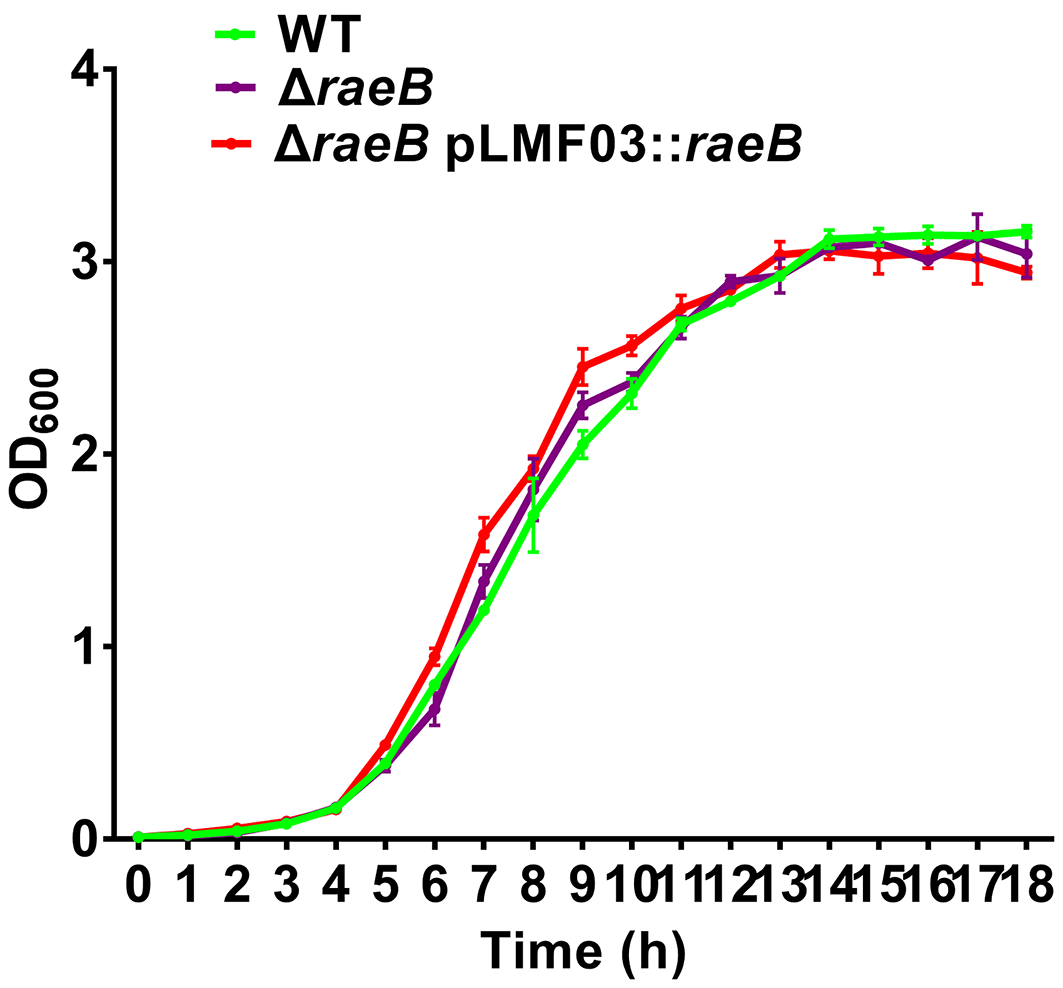

Supplement: Supplementary file 1 [file Image_1.TIF]

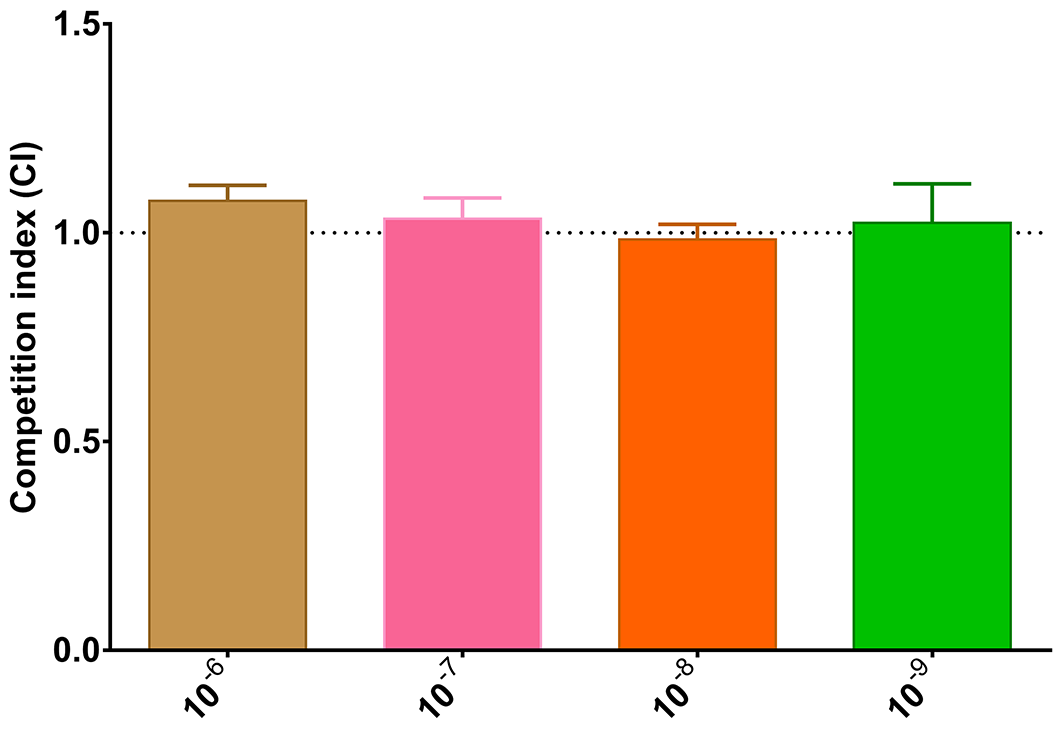

Supplement: Supplementary file 2 [file Image_2.TIF]

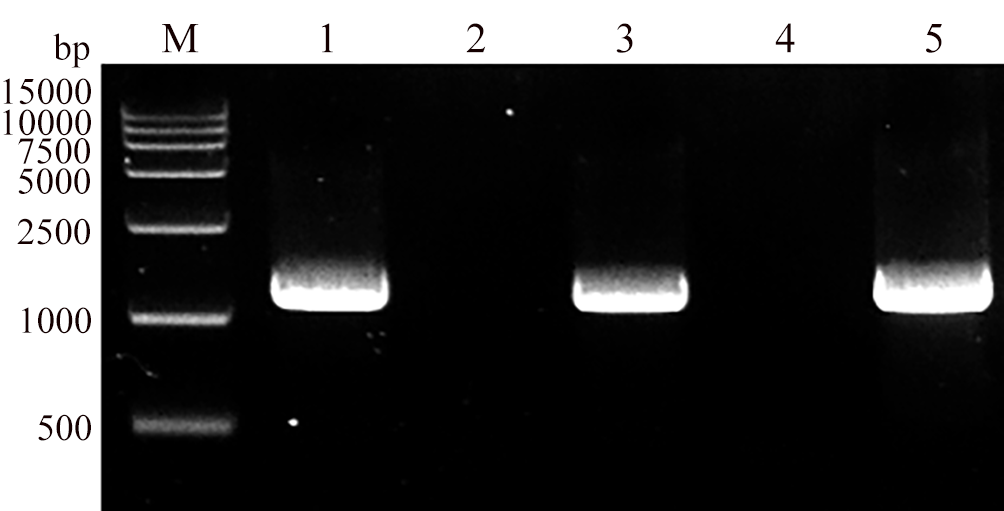

Supplement: Supplementary file 3 [file Image_3.TIF]
