# Supplementary material for: Contribution of RaeB, a Putative RND-Type Transporter to Aminoglycoside and Detergent Resistance in Riemerella anatipestifer
Source: Front Microbiol. 2017 Dec 8;8:2435. doi: 10.3389/fmicb.2017.02435 (PMC5727081; doi:10.3389/fmicb.2017.02435)
Supplement: Supplementary file 4 [file Table_1.DOCX]

Supplementary Table 1 MICs of various antimicrobial agents for *R. anatipestifer CH-1* strains.

| Antimicrobial agent | MIC (μg/mL) | | |
| --- | --- | --- | --- |
|  | *R. anatipestifer* CH-1 | RA-CH-1 Δ*raeB* | RA-CH-1 Δ*raeB* pLMF03:: *raeB* |
| Ampicillin | 2 | 2 | 2 |
| Carbenicillin | 2 | 2 | 2 |
| Cefuroxime | 8 | 8 | 8 |
| Ceftiofur | 4 | 4 | 4 |
| Cephalothin | 16 | 16 | 16 |
| Cefradine | 4 | 4 | 4 |
| Aztreonam | 256 | 256 | 256 |
| Clindamycin | >256 | >256 | >256 |
| Lincomycin | >256 | >256 | >256 |
| Chloromycetin | 4 | 4 | 4 |
| Florfenicol | 4 | 4 | 4 |
| Erythromycin | >256 | >256 | >256 |
| Azithromycin | 256 | 256 | 256 |
| Nalidixic acid | 256 | 256 | 256 |
| Ciprofloxacin | 2 | 2 | 2 |
| Enrofloxacin | 2 | 2 | 2 |
| Tetracycline | 8 | 8 | 8 |
| Trimethoprim | 32 | 32 | 32 |
| Sulfamethoxazole | >256 | >256 | >256 |
| Rifampicin | 128 | 128 | 128 |
| Vancomycin | 256 | 256 | 256 |
| Acriflavin | 4 | 4 | 4 |
| Ethidium bromide | 0.5 | 0.5 | 0.5 |
| Carbonylcyanide *m*-chlorophenylhydrazone | 2 | 2 | 2 |
